# ROMP Synthesis of Iron-Containing Organometallic Polymers ^†^

**DOI:** 10.3390/molecules21020198

**Published:** 2016-02-06

**Authors:** Ileana Dragutan, Valerian Dragutan, Petru Filip, Bogdan C. Simionescu, Albert Demonceau

**Affiliations:** 1 Institute of Organic Chemistry, Romanian Academy, 202B Spl. Independentei, P. O. Box 35-108, Bucharest 060023, Romania; idragutan@yahoo.com (I.D.); petrufilip@gmail.com (P.F.); 2 Petru Poni Institute of Macromolecular Chemistry, Romanian Academy, Iasi 700487, Romania; bcsimion@icmpp.ro; 3 Macromolecular Chemistry and Organic Catalysis, Institute of Chemistry (B6a), University of Liege, Sart Tilman, Liege 4000, Belgium; a.demonceau@ulg.ac.be

**Keywords:** iron-containing polymers, ferrocene, sandwich-complexes, ROMP, hybrid materials

## Abstract

The paper overviews iron-containing polymers prepared by controlled “living” ring-opening metathesis polymerization (ROMP). Developments in the design and synthesis of this class of organometallic polymers are highlighted, pinpointing methodologies and newest trends in advanced applications of hybrid materials based on polymers functionalized with iron motifs.

## 1. Introduction

Metal-containing polymers make-up a highly-valued class of hybrid materials appreciated for their unprecedented qualities [1]. Indeed, essential characteristic traits of organometallic complexes such as chemical, thermal, magnetic, optical, conductive *etc.* impart to metal-containing polymers physical-chemical properties that add to the processability, solubility and mechanical behavior of purely organic polymers. [2,3,4,5,6]. To date, a large collection of metal-containing polymers have been made available including innovatory polymeric frameworks [7,8,9], dendritic structures [10,11,12] and multifunctional composites [13,14,15], all recommending these specialty materials for diverse, cutting-edge applications e.g., as catalytic and drug delivery systems, sensing, optical, electronic and magnetic devices, nanomaterials and energy storage [16,17,18,19,20,21,22,23].

As result of the spectacular advancement in this field, a large pool of synthetic protocols have been elaborated to access newly designed organometallic polymers [24,25,26,27,28] bearing a main-group or transition metal core in either their backbones [29,30,31,32] or side chains [33,34,35,36,37]. These appealing procedures involve step- or chain-growth mechanisms and range from traditional polymerization reactions of metal-functionalized olefins and alkynes to chemoselective condensation, substitution, ring-opening and coordination polymerization processes of the appropriate metal-containing monomers [38,39,40,41,42,43]. Many of these developments e.g., “living” anionic polymerization, controlled radical polymerization (ATRP, RAFT, NMP), ring-opening polymerization occur in a precisely controlled and “living” manner, resulting in fully-characterized polymers or copolymers with unprecedented physical-chemical properties. Following the seminal discoveries of well-defined Mo-, W- and Ru-alkylidene complexes [44,45,46,47], olefin metathesis and metathesis polymerization experienced a tremendous progress in synthetic organic and polymer chemistry [48]. Spurred by these advances, ring-opening metathesis polymerization (ROMP) became a well-accepted synthetic tool for preparing metal-tagged polymers and sophisticated organometallic supramolecular assemblies. In particular, ROMP emerged as a better alternative for polymerization of metal-containing cyclic monomers, mainly those with strained rings, to give metal-containing polymers. Success of ROMP arises from the very active and chemoselective metal-alkylidene catalysts employed, compatible with the metal complexes and often adaptive to various functionalities and reaction conditions [45,46]. Moreover, its “living” character permits narrow molecular weight distributions and low polydispersity to be easily attained as compared to other chain polymerization techniques [49,50,51,52]. In addition to using readily accessible monomers and well-defined, robust catalysts, a further asset of ROMP, *vs.* conventional polymerizations, is that this process occurs under mild conditions and leads to homopolymers and block copolymers with monodispersed chain segments and with complex architectures and desired stereochemistry, what is very important for practical uses [53].

This contribution aims at surveying recent developments in the ROMP synthesis of polymers containing iron motifs in the main or in the side chain, mainly reported after previously published reviews on the subject [54,55]. Information on the chemical and physical properties of these materials is also included highlighting the role of ferrocene and iron sandwich complexes known for their low cost, particular robustness, high thermal and photochemical stability, specific coordination pattern, reactivity and redox profiles. These paramount attributes determine attractive applications of this class of organometallic polymers in the modern fields of electrochemistry, biology, sensing, electronic, optic and magnetic devices, nanomaterials, catalysis *etc.*

## 2. Main-chain Iron-Containing Polymers

Incorporation of transition metals in the main chain of organometallic polymers leads to a substantial change of the properties in the resulting organic-inorganic hybrid materials. The nature of the metal embedded into the polymer chain essentially determines the electrical, electrochemical, optical, magnetic, thermal and mechanical properties of the resulted organometallic polymer. On the other hand, the organic component influences key features of the product like microstructure, processability, solubility, and stability. Selection of the metal to be included into the polymer chain and variation in the structure of the organic fragment allow a fine tuning of the physical and chemical properties of the final organometallic polymer in view of satisfying the increasing demands for practical applications. Polymers containing transition metals in the backbone have been prepared by step-growth polymerization (coordination polymerization, electropolymerization, C–C cross-coupling, olefin metathesis *etc.*) or by chain-growth reactions (ring-opening polymerization, metathesis polymerization *etc.*) [50,51,52,53,54]. Of the latter type of reactions, ring opening metathesis polymerization (ROMP), due to its high efficiency and versatility, became an advantageous procedure to prepare main-chain organometallic polymers that contain ferrocene moieties as part of their backbone.

**Scheme 1:**
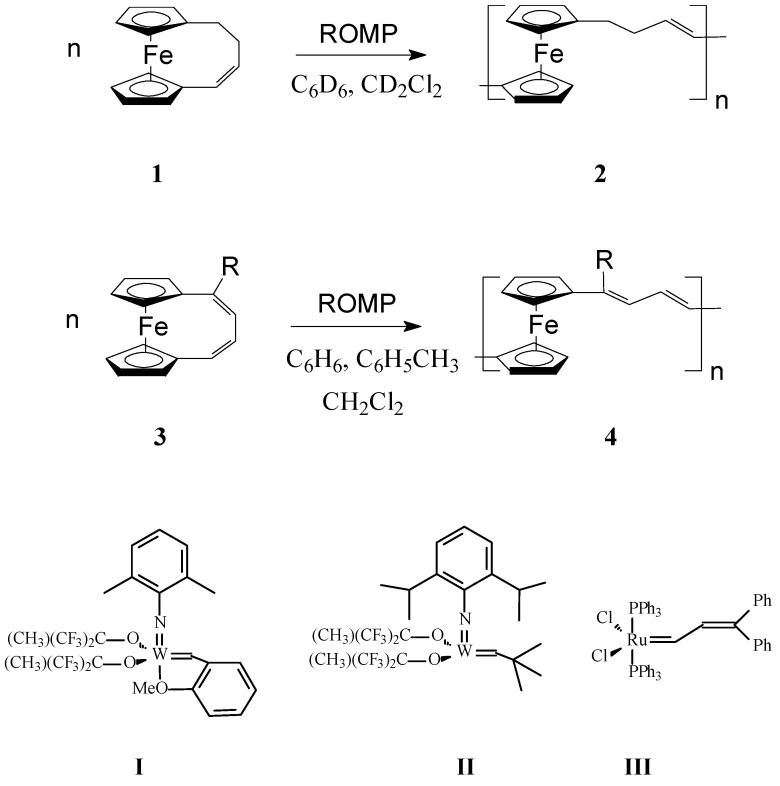
ROMP synthesis of poly(ferrocenylenebuteny1ene) (**2**) and poly(ferroceny1enedivinylene) (**4**) [For **1**: Catalyst **I**, ratio **1**:**I** = 20:1–25:1, concentration of **1** = 0.43 (M); For **2**: Catalyst **I**, ratio **2**:**I** = 25:2–50:2, concentration of **2** = 0.42–2 (M)].

In a very interesting work, Grubbs and coworkers [56] investigated the influence of both ferrocene moiety and the nature of the organic fragment on the metallopolymer properties in ROMP polymers obtained from 1,4-(l,l0-ferrocenediyl)-1-butene (**1**) and 1,4-(l,l0-ferrocenediyl)-1,3-butadiene, (R = H) (**3**) (Scheme 1). Among W- and Ru-carbene catalysts **I**–**III**, catalyst **I** proved to be active with both above monomers giving mainly oligomers. It was inferred that ferrocene linkages in poly(ferrocenylenebuteny1ene) (**2**) and poly(ferroceny1enedivinylene) (**4**) (Scheme 1, where R is H or OMe) would confer flexibility to the conjugated chains by functioning as rotatable π-bonds, affecting thus the solubility and therefore processability of the polymer. Also, the air and thermal stability of organometallic polymers **2** and **4** containing ferrocene units in the backbone should increase since ferrocene is rather stable in air and at temperatures up to 500 °C. In addition, functionalization of ferrocene moiety can be readily manipulated; consequently, many monomers incorporating substituted ferrocene may provide routes to polymers with specific properties. The ROMP of octamethyl-1,4-(1,10-ferrocenediyl)-1,3-butadiene (**4a**) and 1,4-(l,l0-ferrocenediyl)-l-methoxy-1,3-butadiene (**4**, R = OMe), was carried out using the same catalytic system. Whereas a soluble polymer was successfully obtained from the latter monomer, the bulky octamethyl-1,4-(1,10-ferrocenediyl)-1,3-butadiene **4a** could not be polymerized under the above reaction conditions.

**Scheme 2:**
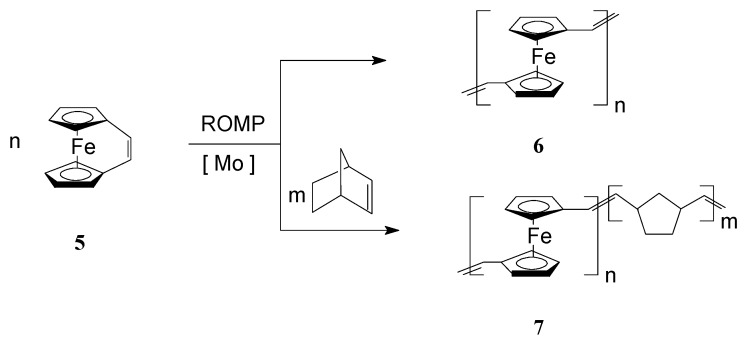
Synthesis of iron-containing polymers and copolymers by ROMP.

To improve the solubility of this class of polymers, Lee and coworkers [57,58] introduced alkyl groups in the unsaturated bridge. Gratifyingly, the substituted ferrocenophane 1,10-((1-tertbutyl)-1,3-butadienylene)ferrocene (R = *t*-Bu), easily polymerized via ROMP, in the presence of W(=CHC_6_H_4_-*o*-OMe) (=NPh)[OCMe(CF_3_)_2_]_2_(THF), to yield soluble high molecular weight polymers (M_w_ = *ca.* 300,000) containing ferrocenylene units in the backbone (Scheme 1, R = *t*-Bu). Variations in the monomer-to-catalyst ratio allowed different molecular weights to be obtained. Remarkably, the polymer exhibited an excellent thermal stability. In another work, Buretea and Tilley [59] successfully performed homopolymerization of *ansa*-(vinylene)-ferrocene and its copolymerization with norbornene using the Schrock Mo initiator Mo(=CHCMe_2_C_6_H_5_)(=NC_6_H_3_*i*-Pr_2_)[OCMe(CF_3_)_2_]_2_ (Scheme 2). Significantly, the homopolymer **6** was insoluble in organic solvents whereas the copolymer **7** with norbornene having M_w_ and M_n_ of 21,000 and 11,000, respectively, displayed a partial solubility.

**Scheme 3:**
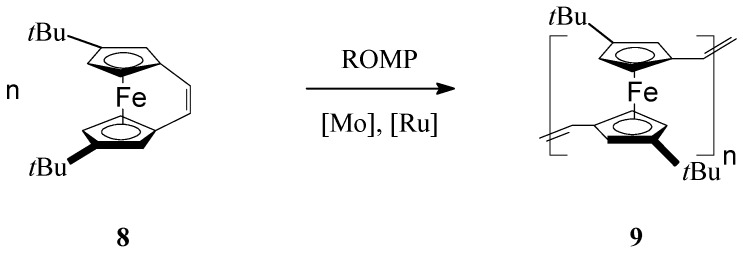
ROMP access to *t*-butyl bearing poly(ferrocenylenevinylene).

By substituting the cyclopentadienyl (Cp) ligands of iron with *t*-butyl groups in *ansa*-(vinylene)ferrocene Fe(η^5^-C_5_H_3_′Bu)_2_C_2_H_2_ (**8**), Manners *et al.* [60] succeeded to prepare soluble *t*-butyl bearing poly(ferrocenylenevinylene) (**9**) via ROMP reaction in the presence of Mo Schrock-type or Ru Grubbs-type catalysts (Scheme 3). It should be outlined that the photolytic and thermal ring-opening polymerizations of this monomer were not successful, and only unreacted starting material was isolated in these processes. UV-vis spectroscopy of the ROMP polymer showed a moderate electron delocalization evidencing the presence of *t*-butyl group in the organometallic polymer. A negative shift of the oxidative potential in the polymer (measured by CV, in CH_2_Cl_2_, on Au electrode, at 22 °C, scan rate 100 mV/s, Δ*E*1/2 = 0.26 V) also was assigned to the electron-donating effect of the *t*-butyl group from the cyclopentadienyl ligands.

## 3. Inter-linked Iron-Containing Polymers

**Scheme 4:**
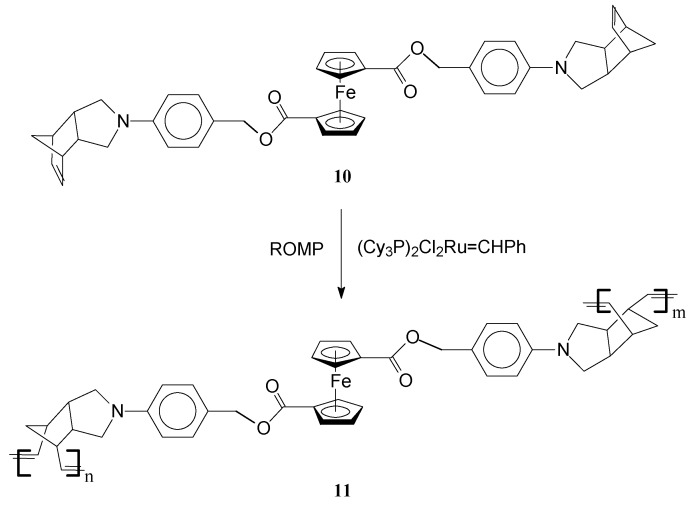
Synthesis of iron inter-linked polymers by ROMP.

Polymerization of the multiple functional ferrocene-derived bisnorbornene monomer **10** with Grubbs first generation Ru catalyst afforded an unusual inter-linked, double stranded helical iron-containing polymer **11** [61] (Scheme 4). Notably, in this process Grubbs catalyst readily induced polymerization of the *endo* pending norbornene units from the ferrocene-derived bisnorbornene monomer. The reaction seems to be facilitated by the slightly flexible ferrocene moiety which assists the second norbornene moiety in adopting a favorable orientation for the polymerization to occur. Moreover, interactions between the linkers of the monomer units give rise to appropriate stereochemical requirements for the self-assembly of the polymer into double stranded helical conformations. Remarkably, the polymer generates DNA-like helices, supercoils, and ladders identified by scanning tunneling microscopy. This feature is very important having in view the potential for biological applications of this type of polymer. It is intriguing that although significant interactions between the functionalities from the linker may occur, the distance (*ca.* 5 Å) between two neighboring Fe atoms is similar to that in the single crystals of the parent ferrocene compounds.

## 4. Side-chain Iron-Containing Polymers

**Scheme 5:**
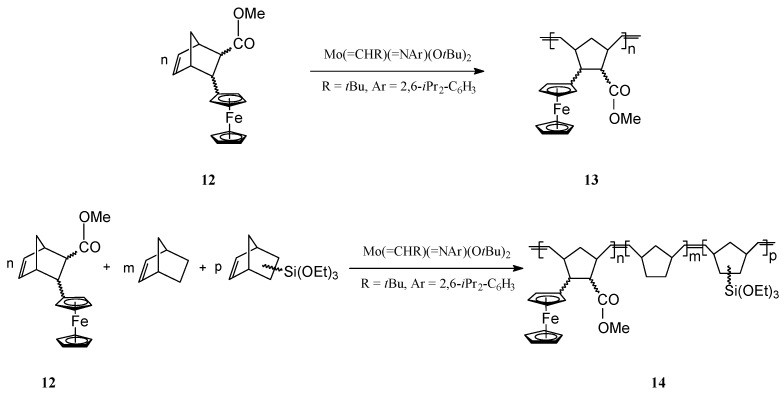
Synthesis of ferrocene-containing homopolymers and copolymers from norbornene monomers.

ROMP of norbornene derivatives substituted with neutral ferrocene groups (e.g., **12**) has been successfully performed by Schrock and coworkers [62,63] using the well-defined Mo catalyst Mo(=CHR)(=NAr)(O*t*Bu)_2_ (R = *t-*Bu, Ar = 2,6-*i-*Pr_2_-C_6_H_3_) (Scheme 5). By this protocol, living polynorbornene polymers **13** and block copolymers **14** bearing ferrocene units in the side-chain have been effectively obtained.

It is significant that with the Mo catalyst a low polydispersity (PDI = 1.05) of the polymer has been attained. The polymer **13** was soluble and displayed fully reversible redox chemistry. When the neutral ferrocene moieties were oxidized to cationic ferrocenium, the polymer became insoluble and suitable for coating electrode surfaces. The process depended on the electrolyte, the polymer molecular weight and polymer microstructure and could be tuned by playing on the size of a non-electroactive block in the polymer [58]. Copolymers with additional functional groups (e.g., **14**, −Si(OEt)_3_, *etc.*) were also examined for their electroactive behaviour when deposited on electrodes; results showed that the copolymer conformation is flexible enough so that all ferrocene sites may sense the electrode.

**Scheme 6:**
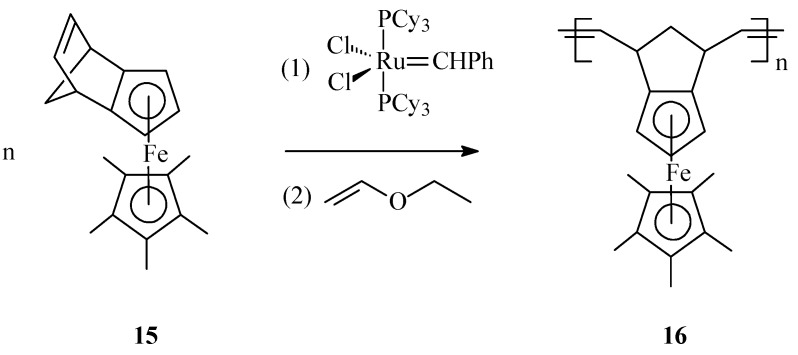
Synthesis of iron-containing polymer **16** by ROMP with Grubbs 1st generation catalyst.

A new type of ferrocene-containing polymers **16**, displaying a totally different microstructure, has been synthesized by Wang *et al.* [64] through ROMP of the norbornene-embedding monomer, η^5^-pentamethylcyclopentadienyl-(η^5^-*exo*-tricyclo[5.2.1.0(2,6)]deca-2,5,8-trien-6-yl)iron **15** in presence of Grubbs first generation catalyst (Scheme 6).

In this organometallic polymer the ferrocenyl units are distributed in a denser environment along the polymer chain what might affect product solubility and processability, but their sterical arrangement may confer advantageous thermal, electronic and magnetic properties.

**Scheme 7:**
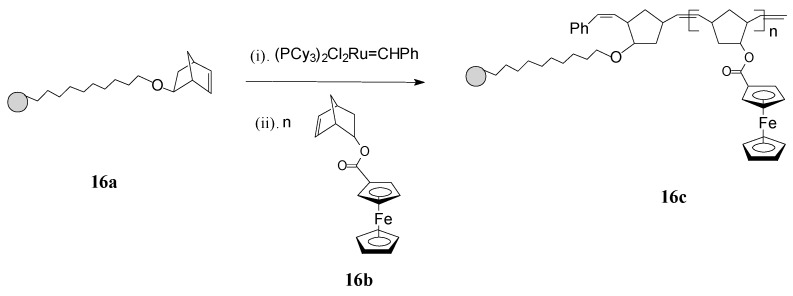
Synthesis of ferrocene-containing homopolymers attached to gold nanoparticles (**16c**) using Grubbs’ 1st generation catalyst.

ROMP has been profitably applied by Nguyen and Mirkin [65] for obtaining new ferrocenyl-substituted polymers anchored on gold nanoparticles (GNPs) with chemically tailorable shell properties. This original method involved: synthesis of 1-mercapto-10-(*exo*-5-norbornen-2-oxy)decane as a linker having a ROMP-able *exo*-norbornene moiety; immobilization of the linker, containing norbornene active sites, on 3-nm Au particles (**16a**); addition of the Grubbs’ 1st generation ruthenium catalyst which thus becomes immobilized on the linker; and, finally, ROMP of the norbornene monomer **16b**, containing ferrocenyl groups, initiated by the Ru catalyst immobilized on the GNPs (see Scheme 7). By this protocol, the neutral ferrocenyl-containing homopolymer **16c** is grown on the GNP in a controlled manner.

**Scheme 8:**
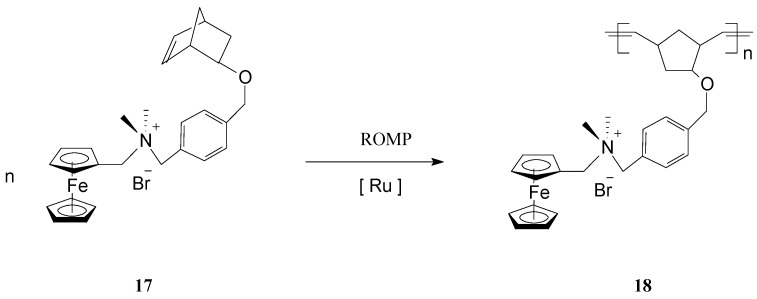
ROMP access to iron-containing amphiphilic polymers.

The polymerization process has been terminated irreversibly by addition of ethyl vinyl ether. By the same procedure, block copolymers attached to GNPs were prepared using two different ferrocene-containing monomers initiated on the GNP with Cl_2_Ru(PCy_3_)_2_=CHPh. Analysis of this type of polymers by transmission electron microscopy (TEM) indicated hybrid particles as having *ca.* 3 nm in diameter, supporting the fact that block copolymerization of two comonomers did not influence the molecular weight of the product. This methodology opens the way for preparing other building blocks and inorganic nanoparticle templates and assemblies, as well as optically active or electroactive norbornenyl-derived materials with perspectives for applications in electrochemically-based diagnosis. For diagnostic applications, the metallopolymers and core-shell nanoparticle-based materials resulting therefrom need to be water soluble, especially when biological molecules are the potential targets. When used in such applications, these polymers can give rise to large, detectable signals that are based on their intrinsic electrochemical, fluorescent, or magnetic properties. In this connection, Nguyen, Mirkin *et al.* [66] have ingeniously achieved the synthesis and characterization of an amphiphilic ROMP monomer **17** that assembles into a single starting material a water soluble ammonium salt, a ROMP-able norbornenyl group and a redox-active ferrocenyl moiety (Scheme 8).

Owing to its amphiphilic character, the monomer was first polymerized to **18** in organic media, then the polymer was dispersed in aqueous solution leading to nanoparticle formation. The solubility of the monomer in different solvents enabled the polymerization to be conducted using the commercial, organic-soluble Grubbs 1st generation catalyst. Also, because the resulting redox-active polymers display a good solubility in polar organic solvents (DMF, MeOH), synthesis could be readily performed in these solvents. It is worth mentioning that this strategy can be extended to many other monomers with ammonium functionalities, capable of forming polymers with structurally programmable parameters. Such metallopolymers are superior candidates to be exploited in electrochemically-based diagnosis.

**Scheme 9:**
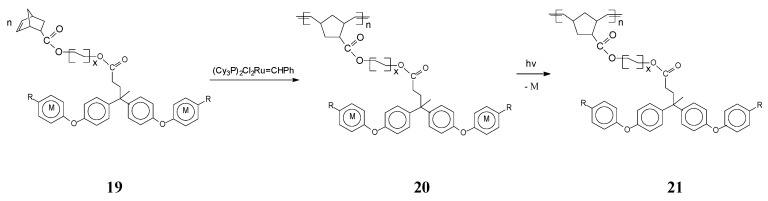
Synthesis of organoiron polymers with different aliphatic spacers. R = Cl, x = 1–5 and M = CpFe^+^ PF_6_^−^.

A broad family of polynorbornenes bearing in the side chain cationic cyclopentadienyliron moieties coordinated to arenes has been communicated by Abd-El-Aziz and coworkers [67]. ROMP of ionic monomer **19** induced by the Grubbs’ catalyst (PCy_3_)_2_Cl_2_Ru=CHPh (monomer/initiator ratio of 20/1) gave, in moderate to good yields (70%–81%), organometallic polymers **20** with the ionic complex evenly distributed in the side chain. The product yield and chemoselectivity essentially depended on the nature of the arene moiety Ar (C_6_H_4_, C_6_H_4_-C(Me)_2_-C_6_H_4_ or C_6_H_4_-C(Me)_2_-C_6_H_4_-C(Me)_2_-C_6_H_4_) and the R substituent (H, Me). These ionic organoiron polymers are soluble in polar organic solvents (such as dimethylacetamide, dimethylsulphoxide and dimethylformamide). Photolytic decoordination of the cationic metal fragments CpFe^+^ PF_6_^−^ from the organometallic polymer **20** led to organic counterparts **21** with higher thermal stability. Interestingly, the thermal stability could be further improved by incorporation of bulkier aromatic ether groups (phenyloxy, naphtyloxy or biphenyloxy) or rigid aromatic substituents in the polymer side chain. Additional investigations have been performed to upgrade the polymer solubility and thermal stability by including aliphatic spacers of varying length within the monomer [68] (Scheme 9).

Remarkably, longer aliphatic spacers within the monomer **19** induced no significant changes on the polymer molecular weight which spanned between 18,000 and 48,000. Important for practical uses, the thermogravimetric analysis showed two successive weight losses, with the first between 204 and 260 °C corresponding to release of metallic moieties and the second, at 368–512 °C, assigned to polymer degradation.

Colored cationic organoiron polynorbornenes functionalized with azobenzene chromophores and prepared by ROMP with Grubbs’ 1st generation catalyst have been reported by the Abd-El-Aziz group [69]. These new hybrid materials consistently integrate photophysical and redox properties and are potentially of interest for photonic devices. Ingenious spacer design, selection of substituents on azobenzene, and position of the organoiron fragment allow playing on the properties of the azo dye polymers. A problematic issue for the team was that the molecular weights could not be measured directly because the cationic organoiron complexes interact when exposed on GPC columns. Fortunately, photolytic demetallation of these polymers permitted isolation of the corresponding organic polynorbornenes whose molecular weights could be determined by GPC. Estimation of the molecular weights of the organoiron polymers was then possible based on the mass of cyclopentadienyliron hexafluorophosphate moieties cleaved from the polymer. Variations in the position and distance of the azo group and organoiron moiety in the side chain demonstrated a reliable correlation between the dye properties and the polymer structure. Novel organometallic polynorbornenes bearing benzothiazole azo chromophores as end entities of the side chains has been later disclosed by the same research group [70]. These polymers displayed a wide range of molecular weights (M_w_ = 24,500–40,900) and two values for glass transition temperatures (T_g_ = 146 °C and 161 °C).

**Scheme 10:**
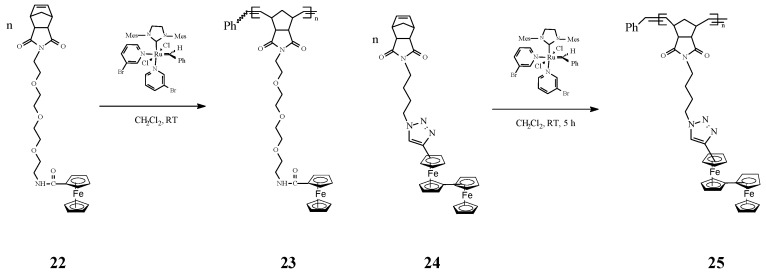
Synthesis of amidoferrocenyl and triazolylbiferrocenyl polymers by ROMP.

An interesting investigation on ROMP homopolymers (e.g., **23** and **25**, Scheme 10) and block copolymers based on polynorbornene containing in the side-chains amidoferrocenyl groups and tetraethylene glycol linkers (e.g., **22** and **24**) was carried out by Astruc and coworkers [71]. The ROMP reaction occurred readily, in a living and controlled manner, reaching nearly 100% conversion at the optimum time and monomer/catalyst ratio, in the presence of Grubbs’ third-generation catalyst. Such polymers have been used to prepare highly stable modified Pt electrodes by progressive polymer adsorption on electrodes, upon scanning around the oxidation potential of the amidoferrocenyl group. The modified electrodes, in particular those prepared from the block copolymers, exhibited sensing of ATP^2^^−^ anions. The authors disclosed that the triethylene glycol branch network in the block copolymers favors the amidoferrocene−ATP interaction by encapsulation. In such a process two amidoferrocenyl groups of the homopolymers interacted with each ATP^2^^−^ molecule. From the reaction stoichiometry the Astruc group suggested a model in which the H-bonding modes are present in the supramolecular polymeric network involving a chelating intramolecular H bond with the β and γ phosphate groups of ATP^2^^−^ and a single H bond between the α phosphate and another amidoferrocenyl group via intermolecular H bonding. A related set of redox-robust triazolylbiferrocenyl (trzBiFc) norbornene-derived ROMP polymers bearing the organometallic group in the side chain have been also synthesized by the same research team [72] using Grubbs’ 3rd generation catalyst (Scheme 10). Oxidation of these polymers with Au^III^ or Ag^I^ gave nanosnake-shaped networks. Further on, these products easily provided modified electrodes that sense ATP^2^^−^ via the outer ferrocenyl units and Pd^II^ via the inner Fc units of the side-chain.

**Scheme 11:**
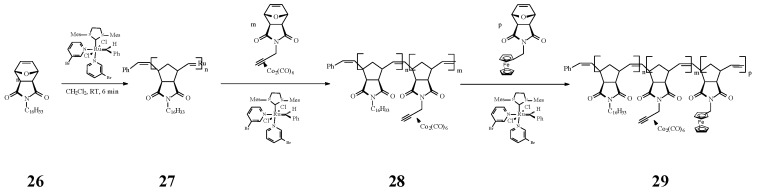
Iron- and cobalt-containing copolymers by ROMP approach.

An intricate ROMP synthesis of heterobimetallic block-random copolymers (BCP) was reported by Tew and coworkers [73] using the Grubbs’ 3rd generation Ru catalyst. They effectively obtained the copolymer **29** consisting of a block homopolymer bearing an alkyl group (C_16_) and a random block copolymer with a pending cobalt complex (Co) and a ferrocene (Fe) moiety (Scheme 11).

Detailed investigations by varying the molar ratio of Co/Fe units indicated that the magnetic properties of the nanostructured BCP materials could be tuned by diluting the cobalt content with Fe entities in the cylindrical domains. Thus, they demonstrated that when the cobalt density decreased, the dipolar interactions of the cobalt nanoparticles weakened, favouring the transition from a room temperature ferromagnetic (RTF) to a superparamagnetic material. These findings have a strong impact on production of new magnetic materials for high density information storage, spintronics, magnetic microelectromechanical systems, and biosensors.

**Scheme 12:**
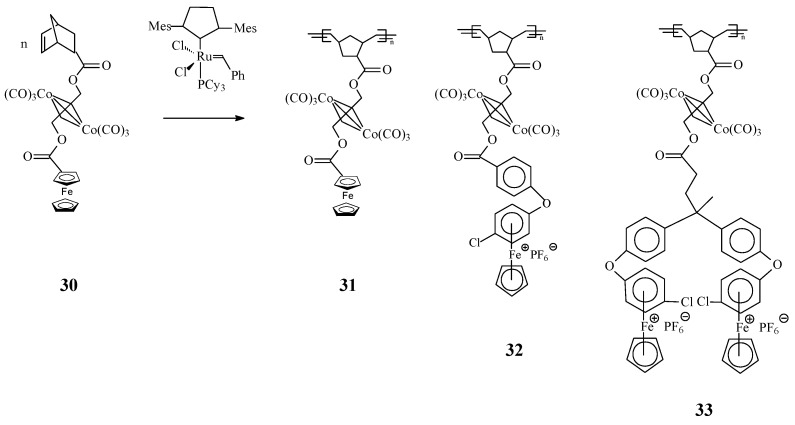
Iron- and cobalt-containing homopolymers by ROMP approach.

Another intriguing trimetallic norbonene complex **30** was designed by Abd-El-Aziz *et al.* [74] by coordination of dicobalt hexacarbonyl to the alkyne moiety of norbornene complex, containing either ferrocene or η^6^-chlorobenzene-η^5^-cyclopentadienyliron hexafluorophosphate. Complex **30** was employed as monomer in the ROMP process to produce polynorbornenes which incorporated ferrocene derivatives and alkyne-bis(tricarbonylcobalt) moieties. Using this type of monomers, organoiron/organocobalt polynorbornenes **31**–**33** with high average molecular weights but moderate PDIs were obtained in presence of Grubbs’ 2nd generation catalyst (Scheme 12).

A remarkable feature in this array of organometallic polymers is the particular effect of the cyclopentadienyl and benzene ligand around the iron core as well as the effect of cobalt on polymer properties. By cyclic voltammetric measurements of both the monomers and polymers at −40 °C, a reversible reduction of the cationic complexes containing η^6^-benzene-η^5^-cyclopentadienyliron and of the dicobalt hexacarbonyl moieties was observed, while a reversible oxidation of the ferrocene containing complex was found. Thermal analysis evidenced that the cobalt carbonyl moiety of the polymers degraded near 130 °C whereas the polymeric backbone was stable up to 350 °C. On the other hand, scanning electron microscopy (SEM) and SEM-EDS showed that the polymers possessed a fine globular morphology and that the distribution of iron and cobalt atoms was homogenous on the macro-scale.

**Scheme 13:**
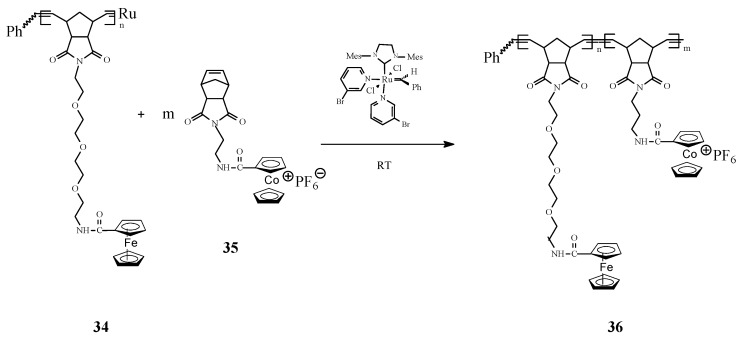
ROMP access to ferrocene/cobalticenium copolymers.

Astruc [75] ingeniously associated ferrocene with cobalticenium motifs in building organometallic block copolymers by living ROMP in presence of the 3rd-generation Grubbs catalyst (Scheme 13). Specifically, the living ROMP of **34** and **35** to ferrocene/cobalticenium copolymers **36** has been performed at a designated theoretical number of monomer units (of 25) for each block. The number of metallocenyl units in each block has been determined from their redox and electrochemical patterns using the Bard–Anson electrochemical method.

**Scheme 14:**
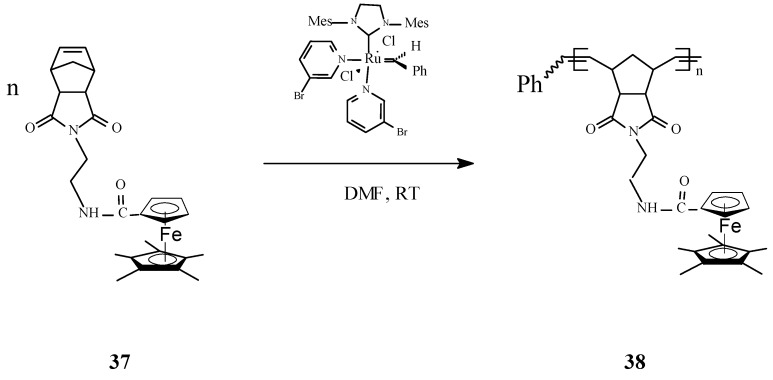
Synthesis of pentamethylferrocene polymers by ROMP.

In a new original work, Astruc and coworkers [76] communicated the synthesis of the first pentamethylferrocene polymers **38** by controlled ROMP of the corresponding norbornene monomers **37** with Grubbs’ 3rd-generation catalysts (Scheme 14). The pentamethylferrocene polymers have been further oxidized to robust polycationic pentamethylferricenium analogues. Also, in this case, the number of monomer units in the polymer has been accurately determined by the Bard-Anson electrochemical method. The redox robust polymers thus obtained allowed to assemble Au, Ag, and Ag^I^ nanoparticles. An important conclusion was that the formation, size and environment of such nanomaterials can be rigorously controlled by single-electron transfer reactions.

**Scheme 15:**
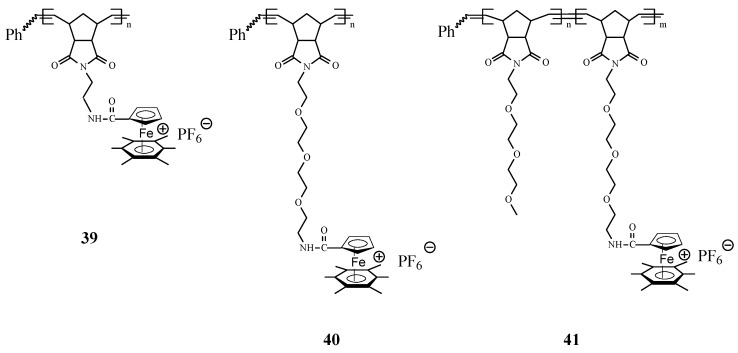
Access to polycationic iron polymers and copolymers by ROMP approach.

Synthesis of interesting polycationic metallopolymers (**39**–**41**) via ROMP of norbornene derivatives containing the complex [Fe(η^5^‑C_5_H_5_)(η^6^‑C_6_Me_6_)][PF_6_], innovatively conceived as an electron supply, has also been successfully achieved by Astruc and coworkers [77] using the 3rd generation Grubbs catalyst (Scheme 15). In this ROMP process, the nature of the amido linker showed to be essential for solubilizing the reaction medium; the shorter trimethylene amido linker required that the polymerization reaction to be carried out in dimethylformamide (DMF) while the longer triethylene glycol amido linker allowed the ROMP reaction to proceed in dichloromethane. The number of the monomer units in the polymer, determined by the Bard-Anson electrochemical method, was found to be close to the monomer/catalyst ratio used in the ROMP. In view of their utilization as stable electron-transfer reagents, it is significant that the redox properties of these metallopolymers, investigated by cyclic voltammetry (CV), exhibited a full reversibility of the Fe^II^ → Fe^I^ reduction wave at −1.35 V *vs.* decamethylferrocene, [FeCp*_2_].

## 5. Branched Iron-Containing Polymers

**Scheme 16:**
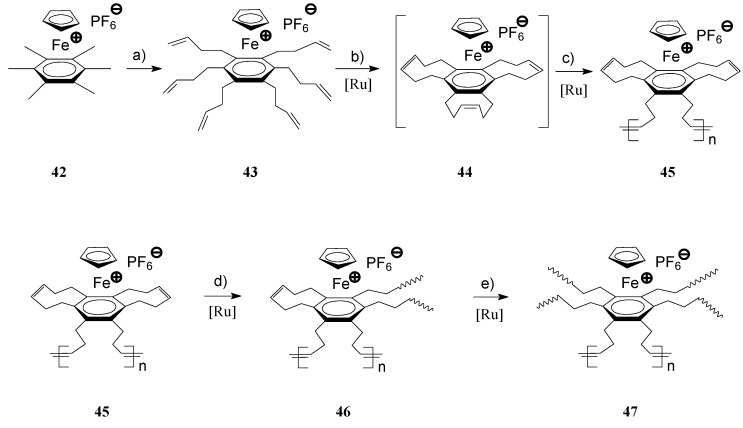
Synthesis of hyperbranched, iron-containing polymers by ROMP [(**a**) Allyl bromide, KOH, DME; (**b**–**e**) Ru(PCy_3_)_2_Cl_2_(=CHPh), CH_2_Cl_2_, room temperature].

The architecturally broad class of hyperbranched, dendritic and star organometallic polymers has recently been the subject of growing interest due to their distinct properties, not available at linear polymers. Along with the progress in transition metal catalysis and particularly in olefin metathesis, synthesis of several intricate hyperbranched and dendritic architectures of metallorganic polymers became possible, circumventing the time-consuming conventional synthetic routes [78,79,80,81]. In their detailed investigations, Astruc and coworkers succeeded to prepare complex **44** by RCM of **43** (Scheme 15, path b) in the presence of Grubbs’ 1st generation catalyst which by successive metathesis steps produced cross-linked polymers **47** containing ferrocene in their recurring units [82,83]. In this complex process, the authors clearly demonstrated that the initially formed linear polymers **45** obtained by ROMP of **44**, reacted further via the second and third cyclodiene units to build the final cross-linked polymer **47** (Scheme 16, path d and e).

## 6. Conclusions

Living ROMP promoted either by Grubbs’ or Schrock’s catalysts proved to be an efficient method for the synthesis of a broad array of organometallic polymers containing iron moieties in the main- or side-chains. The important conclusion here is that both above catalytic systems are compatible with iron atoms from monomers and polymers. Moreover, such polymers benefit from the robustness, coordination pattern, reactivity and redox characteristics of ferrocene and sandwich complexes of iron. The attractive physical and chemical properties of the resulting hybrid materials recommend them for a host of advanced practical applications, presently as fuel cells, organic light-emitting diodes (OLED), magnetic nanomaterials, catalysts, and biosensors.

## Abbreviations

The following abbreviations have been used in this manuscript:

ROMP: Ring-opening metathesis polymerization
Cp: Cyclopentadienyl
RCM: Ring-closing metathesis
ADMET: Acyclic diene metathesis
ATRP: Atom transfer radical polymerization
ATP: Adenosine triphosphate
BCP: Block-random copolymers
CV: Cyclic voltammetry
DMF: Dimethylformamide
GNP: Gold nanoparticle
GPC: Gel permeation chromatography
NMP: Nitroxide-mediated radical polymerization
PDI: Polydispersity index
RAFT: Reversible addition fragmentation chain transfer
RTF: Room temperature ferromagnetic
SEM: Scanning electron microscopy
TEM: Transmission electron microscopy
TrzBiFc: triazolylbiferrocenyl
